# Ethical conference economies? Reimagining the costs of convening academic communities when moving online

**DOI:** 10.1111/geoj.12557

**Published:** 2023-11-20

**Authors:** Michelle Bastian, Emil Henrik Flatø, Lisa Baraitser, Helge Jordheim, Laura Salisbury, Thom van Dooren

**Affiliations:** 1 University of Edinburgh, Edinburgh, UK; 2 Department of Philosophy, Classics, History of Art and Ideas, University of Oslo, Oslo, Norway; 3 Department of Culture Studies and Oriental Languages, University of Oslo, Oslo, Norway; 4 Birkbeck University of London, London, UK; 5 Department of English, University of Exeter, Exeter, UK; 6 Department of Gender and Cultural Studies, Sydney Environment Institute, University of Sydney, Sydney, New South Wales, Australia

**Keywords:** academic inclusion, carbon accounting, community economies, online events, sustainability

## Abstract

Online conferences are widely thought to reduce many of the costs of convening academic communities. From lower carbon emissions, lower fees, less difficulty in attending (particularly for marginalised researchers), and greater accessibility, virtual events promise to address many of the issues that in-person events take for granted. In this article, we draw on a community economies framing from geographers J.K. Gibson-Graham to argue for centring the work of convening within efforts to explore reparative possibilities within the academy. Reflecting on the changing costs arising from moving an originally in-person conference series online, we argue for embracing the opportunities offered. We explore how organising teams might enact alternative values through allocating the material, financial and labour resources traditionally spent for these events differently. We look particularly at how our carbon and financial costs changed, and how, by retaining a fee, we were able to allocate our budgets in ways which redistributed the surplus to participants in need (rather than bolster conference centre profits). We then explore what these changing costs meant in terms of our attendance levels across career stages and geographical locations. Looking at whether our experiment resulted in increased support for online events, we examine the continued ambivalence felt for the virtual. Finally, while we largely explore the benefits of online options, our last section urges caution over assumptions that this move will result in a more sustainable academia, particularly given the intensifications surrounding high quality streaming video, and suggest that we treat current trends as ongoing experiments, rather than solutions.

## 1 Introduction

The publication of the special IPCC report ‘Global Warming of 1.5°C’ in 2019 consolidated a deepening sense of the need to take action on climate change across a huge variety of sectors. Within academia, renewed attention was brought to international conferences, one of the most carbon-intensive activities taking place in our sector (Neugebauer et al., 2020). This happened at the same time as this article’s authors were in the planning stages of the second in a new international conference series. Led by the Temporal Belongings research network, and bringing together scholars and practitioners interested in the social dimensions of time, our first international conference had taken place in 2018 in Edinburgh.^1^ We had been looking forward to consolidating the series with a second event in Oslo in collaboration with the Lifetimes project at the University of Oslo; the Waiting Times research group at Birkbeck, University of London and University of Exeter; and the Sydney Environment Institute at the University of Sydney.

Given the context, the seemingly straightforward move of bringing people together in person to explore shared topics of interest produced profound uneasiness. Interestingly, addressing this discomfort involved an initial turn to calculation. We wanted quantitative information on how carbon intensive our previous event had been in order to gauge our impacts. We thus shared our data with the University of Edinburgh’s Department of Social Responsibility and Sustainability, and using rough estimates, we found that travel alone would have emitted CO_2_ equivalents similar to 13.5 years of energy use for an average European home, but which we produced for only 3 days of conferencing. Given the urgent need to reduce carbon emissions made plain by the IPCC report, we knew we had to pause for a rethink. So, taking up the most prominent alternative circulating in academia, we announced in July 2019 that our upcoming conference would now be virtual.

There have been important criticisms of the proliferation of new metrics in academic life, including Samuel Randalls’ concern that carbon accounting turns debates over ‘the good life’ into ‘a mere calculative challenge’ (Randalls, 2011, p. 128). Arguably, we were relatively unreflective in our initial turn to these new calculations for measuring the impact of our event. In this paper, however, we want to demonstrate how a focus on costs (broadly conceived) actually enabled us to enter into a more exploratory mode. Indeed, in their discussion of the effects of rearranging ways of life in response to climate metrics and targets, Gibson-Graham (2015, p. 54) emphasise the ability for calculations to create room for asking different kinds of questions around the associated costs and how they fit with our wider values. In our case we found ourselves squarely confronting the dominant cost–benefit analysis that justifies in-person events. This is captured by what has come to be called ‘the coffee break problem’ (Bastian et al., 2022), where the reluctance to meet online is often attributed to a loss of joyful interactions.^2^ Yet at the same time we—as in the academic community—know that conferences are exclusionary on multiple fronts, and thus not joyful for everyone. Even so, the benefits of meeting in person are thought to outweigh these concerns. Pushed by our initial calculative approach to rethink costs in a broader way, we found ourselves upending this balancing of costs and values and paying closer attention to the many issues that had arisen during our first in-person event, beyond its carbon intensity. Our 2018 event had taken place in the context of university industrial action in the UK. Disputes over pensions, but also over precarious employment, discriminatory wage gaps, over-work and falling pay, told of a sector rife with exploitation. Indeed this action is still continuing in 2023. Moreover, attendees had faced a range of difficulties, including around affordability, travel restrictions, and money lost on bookings due to visa refusals. Prompted by a carbon calculation, we were offered the opportunity to reconsider the joy of convening in person, see some of the backgrounded costs more clearly, and to reimagine the potential of what we might call the conference economy; that is, all those circulations of people, ideas, funding, waste, fossil fuel dependencies, infrastructures and more.

Building further on the insights offered by Gibson-Graham (2008) we have found it helpful to frame these reflections via the lens of community economies, or diverse economies, particularly given Lindley’s (2009) challenge to ‘academic capitalism’ and the corporate capture of the conference via sponsorships and keynote speaker choices. In thinking through conference organising in terms of *community* economy rather than academic capitalism, we were particularly struck by Gibson-Graham’s emphasis on taking on ‘the task of changing every day embodied practices of surviving well, distributing wealth, encountering, connecting, and sharing with others’ (Gibson-Graham, 2015, p. 61). What might it mean to rethink conferences as forms of economies, which express and structure who can belong and how? How might academics contribute to surviving well and distributing wealth differently through the way events are organised? We were particularly inspired by their insistence that ‘we can choose to create new discourses and counter-technologies of economy and construct strategic forms of interplace solidarity, bringing to the fore ways to make other worlds possible’ (Gibson-Graham, 2008, p. 623). Indeed Gibson-Graham have pushed academics to think about how to go beyond the kinds of critical analyses we are trained in and to develop new methods that ‘excavate, encounter, and extend reparative possibilities for alternative futures’ (Gibson-Graham, 2015, p. 49). While much work in this vein has focused on research methods (e.g., from extractive to participatory) or on alternative writing forms (e.g., from single author to experimental and collective), here we argue for placing the often backgrounded work of organising, meeting and hosting conferences at the centre of discussions about the possibilities of doing academia differently; that is, how might we engage in what Anne Pasek, Emily Roehl and Caleb Wellum have called ‘administrative activism’, where budgets become resources for ‘unconventional interventions’ (Pasek et al., 2020, p. 18).

In this article, we do not have the space to tackle all these questions, however we do offer practical reflections on our experiences with conference convening and how this aspect of academic life can be a venue for experimenting with Gibson-Graham’s ‘reparative possibilities’ via Pasek, Roehl and Wellum’s ‘administrative activism’. Focusing on the online conference in particular, we first discuss the ways costs have appeared in current literature on virtual conferencing, and highlight some key papers from the surge of reflections arising from conjunction of both the climate crisis and the COVID-19 pandemic. We then make the most of the natural experiment afforded by our two conferences, the 2018 in-person event The Social Life of Time, and the 2021 virtual conference The Material Life of Time. Drawing on anonymised data from registrations, details of our financial costs and estimates of carbon emissions, as well as a post-conference participant survey, we discuss our experience of the shifting costs of conferencing and what possibilities we found for responding to our ethical commitments and values, and where our practices fell short of our aims. In doing so, we analyse a number of aspects that contribute to current discussions in the area, including estimates of our carbon footprint, removing barriers to participation (Raby & Madden, 2021a), and debates around charging fees for online events (Roos et al., 2020). However, in our final section we complicate what we have done so far by pointing to work that troubles the online event as the natural solution to the costs of meeting in-person and advocate for not settling too quickly on any particular alternative.

## 2 Counting The Costs

Pandemic restrictions on travel and in-person meetings have contributed to a huge shift in conferencing habits, and there has been a surge of published articles about the design of, and experiences with, virtual conferences (e.g., Milić et al., 2020; Mubin et al., 2021; Song et al., 2021; Viglione, 2020). These complement debates that were already gaining traction prior to the pandemic, and which have been remarkably interdisciplinary. Indeed there have already been helpful scoping studies and reviews which gather together the lessons described in the literature, such as Rubinger et al. (2020) and Spilker et al. (2020). Taken together, this work pushes us to think beyond quantitative approaches to costs and address broader axiological questions around equity, diversity and inclusion.

Even with the variety of expertise that is brought to bear on the challenge of online conferencing, the motivations cited are consistent. A central concern, prominent particularly within the last decade, is the need to reduce the carbon costs of academic life. One of the outcomes is an increasingly robust sense of which aspects of university life contribute the largest carbon emissions, and here conferences figure prominently. Air travel in general, and intercontinental flights in particular, dominate; indeed, academics figure significantly in the select segment of the world’s population that frequently fly long distance (Hopkins et al., 2019; van de Glind & Gomez-Baggethun, 2023). Ultimately, carbon-friendly conferences are not merely a topic for experimentation and research; they have also been the subject of organising and activism. Consider, for instance, the proliferation of flight-reduction policies that have been instituted or presented in university administrations in multiple locations; and the significant presence of the No-Fly and FlyingLess movements in the academy.

Alongside efforts to respond to the climate crises and adapt to the COVID-19 pandemic, there is broad interest in addressing participation costs. By reducing financial, time, travel and administrative burdens, and frequently charging lower fees, virtual events are seen to enable more people to participate on a more level playing field (Shelley-Egan, 2020). Indeed a greater diversity and volume of attendance is being borne out in the data (e.g., Foramitti et al., 2021, p. 2). Further, online conferencing is seen by disability activists to potentially offer better accessibility (Guckenheimer, 2020), such as when captioning is provided.^3^ Several commenters argue that online conferences can reach audiences both within and beyond the academy, with live streams more easily reaching a wide and diverse audience, and better potential to integrate with social media (Boon & Sleigh, 2020). We note, though, that much is left to be explored when it comes to how informal hierarchies, implicit biases and so forth play out similarly or differently in an online conference space (Niner & Wassermann, 2021). Thus, when rethinking core conference infrastructure in the move to online, the counter-technologies offered appear to promise more sustainable, accessible, inclusive academic futures. As a result, across the current literature, there are many arguing for organisers to challenge their assumptions around the value of in-person meetings, and embrace the possibilities that open up from more experimental approaches.

## 3 Moving Online: Our Case Study

Before discussing the specifics of our experiences, we first describe our two conferences to date and the methods we have used to analyse them. The Social Life of Time ran from 5 to 7 June 2018, and was held at the John McIntyre Conference Centre in Edinburgh. There were 169 attendees over the 3 days, with 41 panel sessions and four keynotes. Our second conference, The Material Life of Time, was a synchronous online event which ran across a 49 h period from 15 to 17 March 2021 (or 16–18 March for those in East Asia and Oceania). We met virtually using a platform developed for interactive online meetings called QiQoChat, which provides a ‘social wrapper’ around sets of Zoom meetings that act as virtual seminar rooms. We also used a spatial video tool called SpatialChat (other examples include Gathertown and Wonder) for socialising. Design and tech support were provided by the UK organisation Improbable (a team of improvisers, theatre makers and conversation facilitators) and QiQoChat. We had 418 attendees, and the event included 201 presenters, 45 panels and five keynotes, as well as workshops, roundtables, exhibitions, film screenings and live music. Talks were recorded and available to registered attendees for catch up afterwards.^4^

In comparing multiple dimensions of the costs associated with each of these events (in both the narrow and broad terms we have set out above), we have made use of data from the registration processes, including information on the types of tickets sold, broad details of the timing of registration, the institutions of attendees, and participation at continent and country-income level. Further, we produced a post-event research survey for our online conference and invited all registered attendees to complete it. The survey comprised 16 questions which asked for a mix of multiple choice responses using a five-point Likert scale and open-ended responses. The open questions asked attendees to share their impressions of the temporal, spatial, social and academic aspects of the conference, as well as providing an open field for anything else the respondents would like to tell us. We received 96 responses, a 23% response rate.

In what follows, we have divided up our discussions of the costs of convening into specific key themes. Our analysis covers the issue of carbon costs and then the shifting ‘conference economies’ offered by moving online. These two issues were both prominent in the survey responses. Since the lower travel and financial costs are thought to lead to more inclusive events in terms of numbers and range of participants, and also potentially enthusiasm for more online events, we subsequently discuss who was able to attend, and attendees’ attitudes to attending similar events in the future. At the outset, however, we want to emphasise the intersecting nature of the costs of moving online, which simultaneously affect affordability, travel time and how attendees juggle other responsibilities. As two of our survey respondents neatly summed up:

Online conferences allow for greater and new forms of participation: I could not have attended this event if it had been in-person in the Northern Hemisphere (cost, time, ethical implications of travel).

(Later-career non-tenured, Oceania)

The experience exceeded expectations. I think the benefits of minimising carbon footprint, the convenience of being able to participate while also not having to leave home/childcare responsibilities etc., and minimising cost likely outweigh the joy of spending a few nights in a new city and making friends over beers.

(Early-career non-tenured, Europe)

Thus, for both of these respondents, changing the cost–benefit calculation of conferencing to centre sustainability and accessibility, rather than in-person exchange, had numerous knock-on benefits and arguably enabled them to act more in line with their own values around ethical travel and other care responsibilities. Crucially, we see the everyday embodied practices of academics being reformed when ‘encounter, sharing and connecting’ (as J.K. Gibson-Graham put it) are rethought via the online model.

## 4 Carbon Costs

As discussed above, when we made the decision to move online, we went back to the data we had available for the 2018 in-person conference to create a baseline for carbon emissions with which to compare our efforts. Given that we did not collect exact travel information for our in-person participants, but did have access to their institutional affiliation, what we present here are ballpark estimates which allow us to have a sense of proportional difference (see Table 1). Thus, we are not piloting methods for true carbon accounting, but would point to work others are making in this area as a very welcome contribution (e.g., Raby & Madden, 2021a). For the carbon costs of our in-person event, we worked with the University of Edinburgh’s Department for Social Responsibility and Sustainability who calculated CO_2_e based on the data we provided. Using the institutional location of attendees of The Social Life of Time as proxies for travel distance, we were given an estimate of 175 tonnes CO_2_e for the 169 delegates.^5^ Note that this was for travel only, and did not include emissions from the use of the conference centre, the catering, hotel accommodation, printing or other resources. Using the same method we also calculated what would have been the emissions for The Material Life of Time if all attendees had travelled to the in-person location of Oslo, arriving at an estimate of roughly 311 tonnes CO_2_e.^6^

In comparison, the rough calculations for our online conference emissions were in the region of 7.4 tonnes CO_2_e. Here we used the framework developed by Grant Faber (2021), which provides a very detailed tool for estimating emissions from virtual events. The tool considers factors such as attendees’ computer usage and data transfer, but also emissions per type of device used, pre-event organisational meetings and even desk lighting. As variables, we included only the number of attendees and number of hours of the programmed conference overall (i.e., 418 attendees × 22 h). We know that all attendees did not attend all sessions, and that in addition to the myriad factors considered by Faber, there would have been emissions from attendees viewing the catch-up videos on YouTube (227 h of watch time). Even so, given that some attendees certainly only came to one or two sessions (i.e., 3–4 h vs. 22 h), it is more likely that we have over-rather than under-estimated our emissions. With this figure of 7.4 tonnes CO_2_e, the emissions for our online event were in the region of 2% of the estimated in-person travel to Oslo, or 4% of our actual in-person conference in Edinburgh. Further, even if as Freitag et al. (2021) suggest, the carbon footprint of information and communication technology (ICT) use is systematically underestimated ‘possibly by as much as 25%, by failing to account for all of ICT’s supply chains and full lifecycle’ (p. 1), their suggested margin of error would still only increase our rough estimate to 9.25 tonnes CO_2_e, which is 5% of our in-person event estimate, or 3% of estimated in-person travel to Oslo. Thus, while we would reiterate that these figures are rough, at the same time they initially suggest a significant decrease in environmental impact for the event.

## 5 Financial Costs And ‘Administrative Activism’

Next, we explore the effects that our move to an online event had for the broader sense of conference economies we are proposing in this paper, including finances. In particular, we are interested in the new affordances offered for responding differently to ethical questions around inclusivity, affordability, accessibility and expectations of unpaid labour. Indeed we found quite significant shifts in how we allocated our budget when moving online. Currently, there is significant support for making online conferences free (e.g., Rich et al., 2020; Robinson et al., 2020, p. 578; Roos et al., 2020, p. 3); however, we advocate for a retention of fees in part so these ethical possibilities can be more fully explored. Since it is easy to set up a small Zoom seminar by oneself, there has been the sense that attendees should not have to pay for something that has no physical presence. As a delegate to an Association for the Study of Animal Behaviour 2020 online conference wrote ‘Conference organisers are not usually paid for their work, so what would the fee go towards? No room bookings, food, socials to provide…’ (Raby & Madden, 2021a, p. 3649). This quote epitomises some of the lack of imagination around conference economies that we are keen to challenge. Our experience suggests that there is no need to retain a business-as-usual model that does not pay the, often precarious, members of the academic community who perform such crucial services. Just as the online shift affords the opportunity to rethink the format of how knowledge communities meet, something much discussed in online event literatures, an approach that also highlights the administrative side of events as a site of activism could bring the fore questions of the fee, and what it is put to use for. In our case, freed from the huge costs of booking a university conference centre, and by retaining a small fee, we were able to make our event more inclusive and ethical on a number of fronts.

### 5.1 Setting the fee

Where the decision has been made to charge a fee for online events, there is much confusion around what it should be. Interesting findings have potentially suggested a greater willingness to pay as the pandemic has gone on (Falk & Hagsten, 2022, p. 200). In our case, our conference rates were substantially reduced compared with our in-person conference. Our Edinburgh conference charged fees of between £140 and £240 (with the lower fees for early-bird purchase, lower waged, etc.). This income was on top of support provided by the Wellcome Trust via the Waiting Times project. Our budget was dominated by the conference centre fees, with further costs for keynote travel, accommodation and evening events. While this fee range is on the lower end compared with many conference series (particularly when compared with the sciences), we still heard from many potential participants who simply could not afford this along with their travel costs.

In contrast, our online event offered three options of £10, £30 or £50 and we asked attendees to simply pay what they felt comfortable with, no questions asked. In itself, this move shifted the community economy of conferencing, with questions about fees moving away from predetermined assumptions around who should receive a discount, to attendees using their own judgement about what they felt able to contribute to the collective endeavour. Around 17% paid £50, another 19% paid £30 and 64% of our registered participants paid £10. Far from finding that any fee would appear too high, we received a range of unsolicited comments in the free-text portions of the survey praising the conference’s affordable options. For example;

I really appreciated the affordable tarif[f] to participate, which made the event ethical and truly democratic. It is all to[o] often the case that independent scholars (artists etc.) are priced out of conference participation when their field of praxis is the subject matter, creating a deeply problematic hierarchy.

(Later career non-tenured, Oceania)

This adds a useful data point to research by fellow conference organisers such as Raby and Madden (2021b), who found that their attendees, when asked what they would be willing to pay in a hypothetical scenario, quoted a similar range to our fee structure of up to £50. In our case, we show how these preferences played out in actuality. In practical terms, since only a minority actually paid the higher amounts, organisers adopting our approach should choose their fee structure assuming that most will pay the lowest amount. More detailed research, however, would likely aid in predicting registration payments on the basis of conference attendee demographics. In our case, for example, it is perhaps significant that the majority of attendees were graduate students and early career researchers (discussed further below). In thinking of costs in broader terms though, we see from the quote above that reasonable fee structures can still be seen as affordable, and even enabling more inclusive and less hierarchical events.

### 5.2 Allocating budgets

In terms of how our budgets shifted between the two events, we note that, like our in-person conference, our online event had funding in addition to income from fees, this time from Lifetimes, Waiting Times and the Sydney Environment Institute. This covered keynote fees, tech facilitation and website design support and some platform costs. These costs are relatively straightforward, but in paying for tech facilitation we already see a difference to most humanities or social science conferences which, in our experience, delegate this task to the conference organisers or volunteers who then miss out on participating in the event. We were further able to include expenses that many have overlooked in research on virtual conferences. For example, Rubinger et al. (2020, p. 3) mention that fees freed up from in-person costs, such as the conference venue, can be put towards even higher profile keynote speakers than usual, while others talk about platform costs. We would push for more imaginative uses, which again do not replicate hierarchical conference economies where prestigious speakers are paid more (arguably continuing with a form of ‘academic capitalism’ that Lindley (2009) has critiqued), but where more ethical forms of distribution are considered.

In our case, with the full budget available, we could also pay for support that would have been impossible previously. For example, we offered signing or captioning services to anyone who needed it, live captioning for our keynotes, four paid live music sets (at a time when many UK musicians had not had a gig in over a year due to successive lockdowns), honorariums to all PhD volunteers, and a healthy donation to the UK-based Woodland Trust in lieu of carbon offsetting. Other ideas which we hope to implement in the future include bursaries for attendees needing equipment such as headphones, cameras or data upgrades (see Roos et al., 2020), or childcare support bursaries (Trappes et al., 2020). In this way, elements that have been deemed to be too expensive for in-person conferences—including paying precarious volunteers for their time (cf Robinson et al., 2020, pp. 579–580) and better support for those with access needs—have the potential to be placed at the heart of academic events, even while attendees are paying significantly less.

Further, rather than having minimal choice of venues, as we found with our in-person event, we could also use fees to run our event on a platform of our choosing. We opted for QiQoChat, a platform with ties to the open source movement and dedicated to providing services for dialogue and deliberation. Thus, there are also opportunities for organisers to explore options that challenge the corporate capture of big conferences and provide some independence from the surveillance economy of most big commercial services that are racing to control business opportunities arising from increased online meetings (Zuboff, 2018). Budgets can be leveraged to avoid a passive technological solutionism by simply going with what big tech companies are now providing (see Etzion et al., 2021, p. 2), and the green-grabbing that results when agreements are formed between universities and transnational companies under the guise of supporting less carbon intensive practices (Siamanta & Dunlap, 2019). Additionally, innovative organisations can be drafted in to make the most out of chosen platforms. For ourselves, we worked with Improbable, a UK-based theatre company and open space facilitator, who had already been using QiQoChat themselves to produce inclusive and engaging online events during lockdown restrictions.

So while in some ways the conference participant cited above was right to point out that many of the usual expenses seem to evaporate when moving online, we propose that drawing out the opportunities for the activist approaches advocated by critical geographers *within* conference administration can challenge the hierarchies that were imposed by these usual expenses, and reimagine what we consider to be of value. If funds are freed up by not renting conference venues, we can make events much more affordable, while *also* improving on the in-person conference in terms of enhancing accessibility and putting a wider range of our values into practice. To support participant buy-in, we would recommend that organisers be upfront about costs from the outset, including providing examples of where fees will be allocated. In doing so, we hope for a revaluing of the hidden work of convening and all the many other options we might explore for how we meet in supportive ways.

## 6 Can Reconfigured Conference Economies Build More Inclusive Communities?

So far, we have indicated that in moving online we were able to significantly reduce our carbon footprint, reduce our conference fees, and provide more support to attendees, organisers and others recruited in to design and put on the event. We now want to unpack what this meant in terms of changes to who participated in our events and whether there was enthusiasm for retaining the new model.

### 6.1 Who was able to attend?

As we noted above, a common assumption in the literature is that the reduced costs associated with moving online can expand the number and diversity of attendees at academic conferences, while potentially improving the accessibility of conferences for marginalised communities of scholarship, many of whom are located in the Global South. Hoping to be more inclusive than other synchronous online events that exclusively use the time zone of the host country, our timetable was set up to provide ‘blocks’ of sessions suitable for attendees across the globe, pairing multiple continents together to allow international exposure and networking, using an approach which we analyse in more detail elsewhere (Bastian et al., 2022). As we will show in this section, the move online allowed us to significantly increase the number of attendees and made it easier for precarious scholars to attend. However, we also show that moving online, in and of itself, did not make our event more geographically or geopolitically inclusive.

#### 6.1.1 Attendance levels

Simply in terms of how many people signed up for our event, the online option gave us a big advantage in that we went from 169 registrants to 418, a 247% increase. However, as many have found over the last two and a half years of online events during the pandemic, high registration levels does not always translate into high or even average attendance.^7^ Given the changing patterns of attendance when moving online, we actively encouraged non-presenter registrations in order to ensure healthier audience numbers. This meant that our ratio between presenting and non-presenting attendees was significantly different across the two conferences. While at our in-person conference 4% of attendees registered after the deadline for presenters, for our online event it was 25%. Even so, 68% of our survey respondents said they attended somewhat less or much less than a normal in-person event, and this was coupled with the fact that we still saw some difficulties with audience numbers in the sessions that catered to locations where we had lower registrations. Practically then, we would recommend that in order to retain the experience of the conference as a community of scholars feeding back on papers, organisers look to maximise registrations for their events, such as with direct marketing to, and/or a lower fee for, non-speakers.

Another metric around attendance, which we have not yet seen discussed in the literature explicitly, but which could be useful to keep track of, is how the move to online affects drop-off rates of accepted presenters. In our case while at our in-person conference around a third of accepted papers were withdrawn by the cut-off point for registration, all our accepted participants registered for the online conference, with only a few unable to present on the day due to unforeseen circumstances. This absence of drop-outs meant significantly less churn in the programme, making it more reliable for attendees and less work for the organisers. But also again suggests that the move to online creates more opportunity to attend for interested participants who could not attend the in-person event (for whatever reason) and so had to withdraw.

#### 6.1.2 Attendees’ career levels

In terms of where attendees were in their careers, there was a broad shift away from more established scholars to greater participation from scholars in early career positions, those in more precarious shorter-term contracts, and those outside of academia. Our post-event survey for our online conference indicated that our respondents were largely in their early career stages, with 29% students (MA or PhD), 36% in early career positions and only 25% identifying as later career scholars. Another 9% did not identify with these categories or were not academics. In terms of levels of precarity, we found that 57% of survey respondents who identified as academics were in short-term positions (non-tenured). Other data available to us, which also support this shift, are in terms of the registration levels. Here we do not have directly comparable data, since our in-person fees were described as ‘general’ and ‘student/low waged’, and our online fees were only described by their cost. However, if we roughly compare those who paid £50 or £30 with our ‘general delegates’ and those who paid £10 with our student/low waged delegates and day delegates, as shown in Table 2, then we see a reduction from 58% to 36% for those paying at the higher end of the fee bands and an increase from 42% to 64% for those on the lower end.

#### 6.1.3 Attendees’ geographical location

The potential of the virtual event to overcome barriers to participation based on income, access to mobility and inequalities of power has been much lauded. However, as geographer Tim Cresswell notes, movements of people are always ‘products and producers of power (and thus their attendant inequities)’ (2006, cited in Nevins et al., 2022, p. 235). Our findings suggest that, in the absence of direct relationship building with attendees in areas under-represented in a community of scholars, the simple fact of moving online does not change where attendees come from. Decolonisation, in other words, requires more active work than a simple move online (see Bruno & Faiver-Serna, 2022). As Table 3 demonstrates, there was very little change in where our delegates came from, either in terms of country income levels, or geographically, aside from the increased participation from participants at institutions in the Oceania region.^8^ While this may have been due to the ease of attending without an expensive long-haul flight, it probably also reflects that the team for the second Temporal Belongings conference included an organiser from Australia who could recommend the event to their networks. These findings suggest that without specific commitments to relationship building, redistribution of resources, and ensuring diversity in the organising team, the provision of an online space will not in and of itself ameliorate the lack of inclusion of majority world countries. Instead structural hypocrisies and over-representation of academics from the Global North remain, and the naive optimism of hoping to overcome the inertia of exclusions through tech solutions alone is laid bare (de Souza, 2019; see also Graham, 2013).

## 7 The Value Of Experimentation and The Social ‘Costs’ Of Moving Online

For J.K. Gibson-Graham, part of the work of experimenting with diverse economies is an expansion of experience and of learning to be affected differently. They note that as participants in these experiments ‘break with old habits and try out new ones, their range of experiences increases as do their connections with their own bodies, other people, technologies…’ (Gibson-Graham, 2015, p. 53). As we have argued elsewhere (Bastian et al., 2022), a primary goal in organising The Material Life of Time was to experiment with the creation of an online, convivial event across time zones, in order to compensate for the loss many associate with moving away from physical conferences, especially the valued experiences of embodied, face-to-face joyful interaction. The kind of value generated through experiments like these is somewhat incongruous to most discussions of costs; and yet, this notion of value was central to the choices we made, and to the willingness of participants to be involved in similar experiments in the future.

Feedback from current experiments can only gesture towards what a transitioned future might look like, indeed only the cumulative effect of efforts to break with old habits may amount to a cultural shift. However, the data we do have indicate that, while most of our respondents still had preferences for in-person events, the experience of the conference shifted many of their senses of what was possible. Indeed, perhaps the most encouraging (and surprising) result from our survey was that as well as reducing carbon we may have also increased joy; that is, when asked ‘On a scale of 1–10 how much did you enjoy the conference?’ attendees ranked their overall enjoyment of the online conference slightly *higher* than attendees from our in-person event in 2018 (an average of 8.5/10 vs. 8.4/10). Yet at the same time, this high satisfaction, and the widespread awareness of the environmental benefits of the online model, did not translate into unequivocal support (see Table 4). When asked about preferences for the format of the conference post-pandemic, respondents could pick any of the three options proposed, resulting in 31% supporting fully online, 42% hybrid and 27% in-person, with 38% of respondents selecting hybrid as their only option. This higher preference for the hybrid option supports similar findings from Rissman and Jacobs’ (2020) survey of perceptions of conferencing post-pandemic. We extend this survey by showing that even after attending an online event that most were very happy with, hybrid events were still the most preferred option.

For those committed to in-person events only, key reasons were the perceived need for face-to-face interactions in order to network and create stronger bonds, and second, the difficulty of making time for the event when not physically present. For example:

I know all the environmental reasons against this but some conversations and modes of thinking need face-to-face.

(Later-career tenured, North America)

I will be attend many more sessions, meet more scholars and have much more interactions with those working in the same or similar areas.

(Early-career tenured, Asia)

Even so, others showed how the new experience shifted their sense of what was possible. For example:

I would have enjoyed an in-person meeting much more, but now that I’ve experienced this format, I know that it works, and I know that lots of people can come who wouldn’t otherwise be able to. So, it’s a tradeoff.

(Later-career tenured, North America)

I’m optimistic about the future of online conferences after this experience and feel like it might be worth experimenting with different formats.

(Later-career tenured, Europe)

Thus, while the highly positive experience of the event overall did not lead the majority to prefer online events, the event was able to challenge expectations, draw out new perceptions of the standard cost/benefit assumptions of in-person events, and support willingness to continue experiments in this direction.

## 8 Remaining Aware Of Unintended Consequences

Reconceiving the tradition of technocratic ‘wicked’ problem-solving in a complex world, social theorist Martin Savransky (2021, p. 3) has argued for being alive to the transformative, but ultimately irresolute, potential of grappling with the ever-proliferating problems resulting from our actions. In our case, and following prompts from Anne Pasek and work arising from her Low Carbon Methods group,^9^ we want to reactivate a critical eye at this final stage in our paper on the most prominent alternative to carbon-intensive academic life: the online conference centred on high-quality streaming video. In particular, we return to the intersecting nature of the costs and benefits of virtual events discussed above, including lower costs, more flexibility, and ease of attendance. These features have generally been celebrated, both in the literature and by our own attendees. However, recent literature on the carbon emissions arising from ICT suggests potentially unintended negative consequences.

Video streaming forms the backbone of nearly all online and hybrid conference designs, with Twitter conferences being a key counter-example (Boon & Sleigh, 2020).^10^ This follows a wider trend where video streaming is rapidly increasing as a percentage of internet traffic, particularly since the start of the COVID-19 pandemic (Marks et al., 2021).^11^ As we have shown, streaming offers much lower carbon emissions than an in-person event, however, given the deep cuts in carbon emissions needed to meet the Paris 1.5° targets, ICT needs to replace carbon emissions in other industries while *also* reducing its own outputs (Freitag et al., 2021, p. 23). However, with faster networks making streaming easier, more intensive uses of services arise, further increasing overall emissions (Freitag et al., 2021, p. 2). Indeed research from The Shift Project (2019) suggests that digital technologies account for 3.7% of global carbon emissions and this is rising much faster than previously forecast. While this number seems low, this is actually higher than some calculations of the aviation industry footprint which is thought to be around 2.5% of CO_2_ emissions.^12^ Unpacking these various knock-on effects has thus led to caution over whether impacts are as easy to eliminate as many have assumed.

Crucially, the assumption that virtual conferencing will reduce carbon intensive academic practices only works if online conferences *replace* their in-person counterparts. As Freitag and colleagues write, ‘ICT’s net effect on global emissions depends on the extent to which ICT substitutes more traditional, carbon-intensive activities rather than being offered *in addition* to them’ (Freitag et al., 2021, p. 2). It was clear that this kind of substitution happened frequently during the pandemic. However, there is also the possibility that with resumed ability to travel academics may attend *more* events, by maintaining attendance at in-person conferences and using the affordability and flexibility of online conferences to participate when they are unable to travel or commit to the full time period. Indeed, we have already personally experienced a kind of ghostly doubling of attendance, where we have been giving a talk at an online event, while simultaneously being ‘in person’ at another. Thus the new comfort with online and hybrid events may mean we are left worse off than before. In terms of carbon outputs, but also in multifaceted ways including the creep of work into so-called non-work hours, or indeed its intensification. Advocates for the move to online need to be wary of increasing expectations for conference attendance and academic networking in regard to promotion, particularly given that increased workloads have contributed to the continuing strike action in the UK that affected our first conference.

Thus, even if, from the perspective of our own series of events, emissions have been reduced by moving our large events online, if our attendees add a new conference that they might not have gone to before because of the savings in time, cost and travel from our event, then we have not reduced academia’s overall carbon emissions. As Rissman and Jacobs point out, ‘it is likely that researchers, like most people in general, will crave in-person interaction once travel feels safe again’ (Rissman & Jacobs, 2020, p. 3). Indeed our own findings hint towards an additive approach, where academics attend more, rather than a substitution approach, where they attend less. For example, after weighing up the advantages of in-person versus online, one survey respondent wrote, ‘This said, some conferences may just be online and others in person. I’d like to do a bit of both’ (early career tenured, Europe). So even while online events start to embed themselves as a recognisable repertoire for academic knowledge exchange, we would argue that conference organisers need to push past the headline deep drops between in-person and online carbon costs. Instead we need to take seriously unintended consequences which will themselves call for further experimentation, such as low carbon IT approaches found in the report *Lean ICT: Towards Digital Sobriety* (Ferreboeuf et al., 2019), or actively constrain our attendance to only a certain number of events per year.

## 9 Conclusion

The need for geographers to shift to low-carbon forms of academic teaching and research (Williams & Love, 2022) has arguably reset ongoing debates in the discipline about the appropriate focus of academic activism ‘out there’ and/or ‘in here’. In this paper, we have centred the administrative aspects of conferences as an important site of activism, alongside research and teaching, and contributed to discussions around its embedding within ‘academic capitalism’. We have sought to move beyond the accounting lingo of easily quantifiable costs and benefits that so often become the unquestioned parameters of green transition politics as well as the accompanying good calculative subject. Instead we have pointed towards the way that counting up carbon costs can lead to highly enjoyable experiments that seek to put ethical and community values into practice. In focusing on both J.K. Gibson-Graham’s work on community economies, and Pasek et al.’s concept of administrative activism, we have been able to engage with a wide set of debates, and argue for their interconnection and relevance in the seemingly banal act of organising a conference.^13^ Asking hard questions about budgets, about who should be paid and how much, which technologies we are being funnelled into by our institutions and how might we resist them, how to centre support needs that are often treated as peripheral luxuries, all these and more become open opportunities for experimentation once conference administration is treated as a site of activism. However, we have also shown that gestures towards the greater inclusivity of techno-fixes, premised on fantasies of an internet accessible-to-all, will not address entrenched inequalities, and may indeed lead to additional carbon costs, work pressures and vectors of exclusion. Calls for a reduction in air travel in academia are thus linked not only with the need to reduce carbon consumption, but must also be connected with attempts to decolonise the academy through revealing the link between ecological injustices, the ‘ontological excess’ of some academics in the wealthy nations and universities of the Global North, and coloniality (Nevins et al., 2022, p. 6).

For J.K. Gibson-Graham, work on reshaping how communities share and exchange resources need not be only a topic of research, but also enacted when academics become ‘directly enrolled in performing alternative economies’ (Gibson-Graham, 2008, p. 626). They advise those taking up the experimental approach to be ‘open to what it has to teach us’ and to recognise that ‘what we are looking at is on its way to being something else’ (Gibson-Graham, 2008, p. 628). In putting together an online conference on the material life of time, we found ourselves also experimenting with the materiality of the conference, and how a shift from a large conference centre to supported video streaming meetings changed the very form of the conference economy. This included the effects of our emissions and our responsibilities for them, the entrenched structures of who could attend and in what ways, and whose labour was going to be more prominently recognised. Even so, we noted that the very things that make an online conference appealing—the lower price, the ease of attendance, the increased number of attendees, the feel-good factor of not flying—could unfortunately work together to increase academia’s carbon footprint, rather than reduce it. In considering the longer term future of virtual events, then, we would do well to keep Marks et al.’s words in mind: ‘Scenarios in which ICT is powered by renewable energy and we can continue to consume all we want are unlikely’ (Marks et al., 2021, p. 48). Thus we argue for recognising online alternatives to in-person events, not as the solution, but as continuing experiments ‘on their way to being something else’.

## Figures and Tables

**Table 1:** Comparison of rough estimated CO2e across our events.

Conference	No. of attendees	Estimated emissions
Social Life of Time (in person)	169	175 tonnes CO_2_e
Material Life of Time (if held in person)	418	311 tonnes CO_2_e
Material Life of Time (online)	418	7.4 tonnes CO_2_e

**Table 2:** Comparison of changes in fees paid across our events.

	General delegates/general admission £50 + general admission £30 (%)	Student/lower waged + day delegates/general admission £10 (%)
Social Life of Time	58	42
Material Life of Time	36	64

**Table 3:** Comparison of attendees across in-person and online conferences by country income levels and continent (may not add up to 100% due to rounding), for those we had institutional affiliation data for (Social Life of Time n = 169, Material Life of Time n = 377).

	Country income (World Bank)					Continent					
	High (%)	Upper-middle (%)	Lower-middle (%)	Low (%)		Africa (%)	Asia (%)	North America (%)	South America (%)	Europe (%)	Oceania (%)
In-person	97	1	2	0		0.5	4	22	1	68	3.5
Online	97	2.7	0.3	0		0.4	1.6	23	2	61	11

**Table 4:** Preferences for future events, *n* = 92.

Conference option	Total preferences (%)	No of respondents (and %) who exclusively preferred this option
Online	31	19 (21%)
Hybrid	42	35 (38%)
In-person	27	10 (11%)

## Data Availability

Research data are not shared, as consent was not received from survey participations for wider dissemination.

## Footnotes

1 See our website for more details: https://www.temporalbelongings.org/sociallifeoftime.html.

2 Note that in the article cited we explore the issue of conviviality and joy at online events extensively, including the technologies we used to support online sociality and conflicts around making time for online events.

3 The tendency to provide automatic captioning, however, has led to the unfortunate proliferaton of ‘craptions’, where the voice recognition software simply guesses at what the speaker is saying (Vasquez, 2021). Automatic captioning that prioritises particular forms of standardised language also disadvantages those whose speech forms and patterns may diverge from such norms. It can also render presentations with bilingual elements inaccessible.

4 We discuss the design side of the event, particularly our emphasis on networking and social activities, in detail in Bastian et al. (2022).

5 Based on the following assumptions: all UK journeys were by rail; all non-UK journeys were by air; all journeys (air and rail) were in economy class; all journeys were direct from the airport/rail station of the city of the Institution to Edinburgh (airport/railway station).

6 Note that the relatively peripheral location of Oslo, Norway will have also had an effect on this high carbon cost. See research on reducing in-person carbon emissions by choosing more centralised transport hubs for conferences (Desiere, 2016, p. 58).

7 Although research has shown that even at in-person events, attendance can still be quite low. See, for example, Rom (2012, pp. 336–337), who suggests that at the American Political Science Association conference in 2005–2006, attendees went to less than 20% of the conference on average.

8 These figures are based on the same location data that we used for the carbon emissions estimates. Also while making use of the categories offered by the World Bank, we are mindful of recent critiques of these and other ‘spatial heuristics’ and all that they cover over (Trauger & Fluri, 2020).

9 More details here: https://www.annepasek.com/low-carbon-methods.

10 See also the Low Carbon Methods group’s zine based conference https://www.diymethods.net/.

11 Thank you to Anne Pasek for pointing us to Marks et al. (2021), as well as Freitag et al. (2021).

12 https://ourworldindata.org/co2-emissions-from-aviation.

13 Here we would also like to highlight others working on approaches such as ‘more-than-research’, which seek to channel the resources given to academics for their work to wider benefit (Hernández et al., 2021, p. 840).
